# *Phlebotomus *(*Euphlebotomus*) *barguesae *n. sp. from Thailand (Diptera – Psychodidae)

**DOI:** 10.1186/1756-3305-2-5

**Published:** 2009-01-08

**Authors:** Jérôme Depaquit, Frédérique Muller, Nicole Léger

**Affiliations:** 1 JE 2533 – USC AFSSA-VECPAR, Faculté de Pharmacie, Université de Reims Champagne-Ardenne, 51, rue Cognacq-Jay, 51096 Reims cedex, Reims, France

## Abstract

**Background:**

A few studies have been carried out on the Phlebotomine sandflies from Thailand. Within the Phlebotomine sandflies, the genus *Phlebotomus *Rondani & Berté, 1840 contains the vectors of leishmaniases in Europe, Africa and Asia. It includes several subgenera. Among them the subgenus *Euphlebotomus *Theodor, 1948 contains at the present time 12 taxa. The type-species of this subgenus is *P. argentipes *Annandale & Brunetti, 1908, the vector of *Leishmania donovani *(Laveran & Mesnil, 1903) in India.

**Results:**

A new species of sandfly, *P. barguesae *n. sp. is described from limestone caves in Thailand. The male-female gathering in the same species is based on ecological, morphological and molecular criteria (homology of mtDNA cytochrome c oxidase I sequences). The inclusion of *P. barguesae *n. sp. in the subgenus *Euphlebotomus *is justified on the basis of characters of the male genitalia (five spines on the style, bifurcated paramere, and no basal lobe on the coxite) and of female pharyngeal armature (two kinds of teeth). It well differenciated from another sympatric species: *P. mascomai*.

**Conclusion:**

The new species described in the present study has smooth spermathecae. This original morphology opens a discussion on the heterogeneity of this subgenus.

## Background

Species of phlebotomine sandflies recorded in Thailand belong to the genera *Phlebotomus *Rondani & Berté, 1840, *Idiophlebotomus *Quate & Fairchild, 1961, *Chinius *Leng, 1987 and *Sergentomyia *França & Parrot, 1920 [1-5]. Three of them belong to the subgenus *Euphlebotomus *Theodor, 1948: *Phlebotomus argentipes *Annandale & Brunetti, 1908, the main vector of *Leishmania donovani *(Laveran & Mesnil, 1903) in India, *P. philippinensis gouldi *Lewis, 1978, found in the rain forest of Thailand, and *P. mascomai *Muller, Depaquit & Léger, 2007, from limestone caves.

## Methods

### Type locality

Thailand, province of Ratchaburi, district of Muang, sub-district of Huay Phai, inside the cave "Khao Tham Khun Chom" (13°48'85,6"N et 99°70'35,7"E). Altitude: 35 m. above sea level.

All the specimens were caught in July 2004 and February 2005 (Barbazan *recoltavit*) by CDC miniature light traps between 5 p.m. and 9 a.m. They were kept in 96° ethanol, and mounted *in toto *for morphological study according to Abonnenc's method [6]: 4–8 hours in 10% KOH solution followed by eight baths (20 minutes each) in water, then at least 1 hour in Berlese's medium. Females were directly mounted in this liquid with their spermathecae dissected, then re-mounted in chloral gum when possible. Some males were dehydrated in ethanol of growing concentrations (from 70% to absolute ethanol) then in beech creosote and finally mounted in Canada balsam.

23 specimens have been studied among them 16 (10 males and 6 females) were prepared for morphological study and seven specimens (5 males and 2 females) were prepared for both morphological and molecular studies. Four topotypes of *P. mascomai *(two males and two females) coming from the same cave have also been prepared for both morphological and molecular studies and processed like *P. barguesae *n. sp. specimens. The selected gene was cytochrome c oxidase I of the mtDNA. The head and genitalia of each sandfly were cut off in a drop of ethanol, cleared in boiling Marc-André solution, and mounted under a cover slip in gum chloral for identification. These slides are available upon request to the corresponding author. Genomic DNA was extracted with the QIAmp DNA Mini Kit (Qiagen, Germany) by following the manufacturer's instructions, except for the crushing of sandfly tissues with a piston pellet (Treff, Switzerland). Polymerase chain reactions (PCR) were performed on a 50 μl volume using 5 μl of extracted DNA solution and 50 pmol of each of the two primers LepF (5'-ATTCAACCAATCATAAAGATATTGG-3) and LepR (5'-AAACTTCTGGATGTCCAAAAAATCA-3') [7] were used under the following thermal profile [8]: an initial denaturation step at 94°C for 3 min, followed by 5 cycles of (denaturation at 94°C for 30 s, annealing at 45°C for 90 s and extension at 86°C for 60 s), then 35 cycles of (denaturation at 94°C for 30 s, annealing at 51°C for 90 s and extension at 86°C for 60 s) and a final extension at 68°C for 10 min. using 0.25 μl of *Taq *DNA (5 prime, Germany). Sequencing was performed on both strands by the dideoxy chain-termination method with the *Taq *dye-terminator chemistry kit for ABI 373A (Perkin-Elmer, Foster City, CA, USA), using PCR primers. Direct sequencing of both DNA strands was performed using the primers used for DNA amplification. The correction of sequences was done using pregap and gap software [9,10]. Their alignment has been performed using Bioedit software [11].

Specimens were observed using a BX 50 microscope (Olympus, Japan). Measurements were collected using the Perfect Image software (ARIES Company, Chatillon, France) by means of a video camera connected to the microscope.

## Results

### Description of the male of P. barguesae n. sp. (Figure 1)

**Figure 1:**
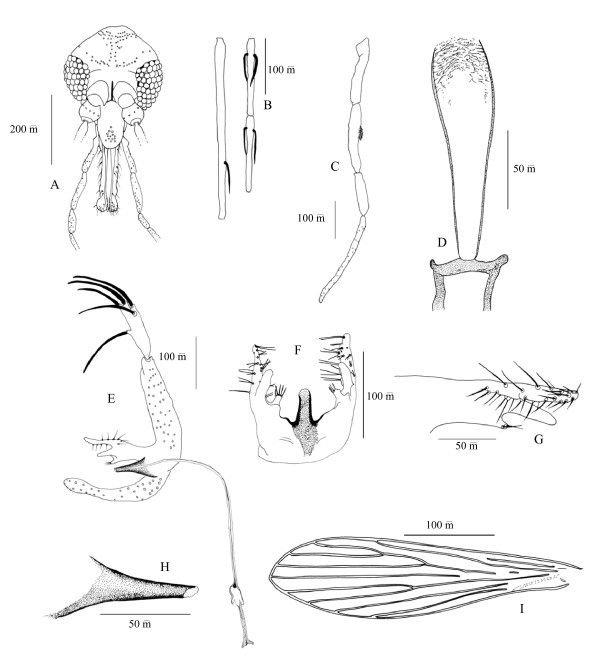
***P. barguesae *n. sp. male**. A: head; B: 3^rd^, 4^th ^and 5^th ^antennal segments; C: palp; D: pharynx and cibarium; E: genitalia; F: aedeagus and parameres (ventral view); G: paramere; H: aedeagus; I: wing.

On the 15 specimens investigated, 8 have been measured.

#### • Head

- Inter-ocular suture: incomplete.

- Cibarium without teeth. No pigmented patch.

- Pharynx: length = 143–157 μm, armed by small teeth at the posterior part.

- Palpal formula: 1, 4, 2, 3, 5. About 10 club-like Newstead spines implanted in the middle of the third palpal article.

- Ascoid formula: 1/III – 2/IV-VIII....(next segments were missing) Ascoids, doesn't reaching the next articulation. AIII = 340–400 μm. AIV = 145–168 μm. AV = 142–165 μm. AIII/AIV+AV = 1.17–1.22.

- Labrum = 175–200 μm. AIII/labrum = 1.9–2.05.

- Eyes consisting of about 45–50 ommatidial facets.

#### • Thorax

- 2–3 antero-inferior setae on the mesanepisternum.

- Wings: length = 1.8–2 mm; width = 410–570 μm. Pi: positive.

- Long anterior, median and posterior legs measuring a similar total length (3.7 mm), without spines.

#### • Genitalia

- Well developed.

- Coxite: length = 160–210 μm, width = 40–60 μm, without any tuft of internal setae. length/width = 3–3.7.

- Style: length = 105–140 μm. Five spines on the distal part: two terminal, one sub-terminal, one intermediate near the sub-terminal and one inner proximal.

- Coxite lenght/style lenght = 1.45–1.75.

- Paramere: trifurcated. Measurements according to Leng and Lewis [12]: superior clasper = 49–69 μm with many setae; medium = 34–37 μm without setae; inferior = 10–13 μm with a group of 4–5 setae. No spine along the aedeagus.

- Surstyle = 178–230 μm

- Aedeagus: short, thick and strong, brown except its blunt-end tip.

- Genital filaments: lenght = 290–380 μm; Genital pump = 105–115 μm. Genital filaments/pump = 2.7–3.1.

### Description of the female of P. barguesae n. sp. (figure 2)

**Figure 2:**
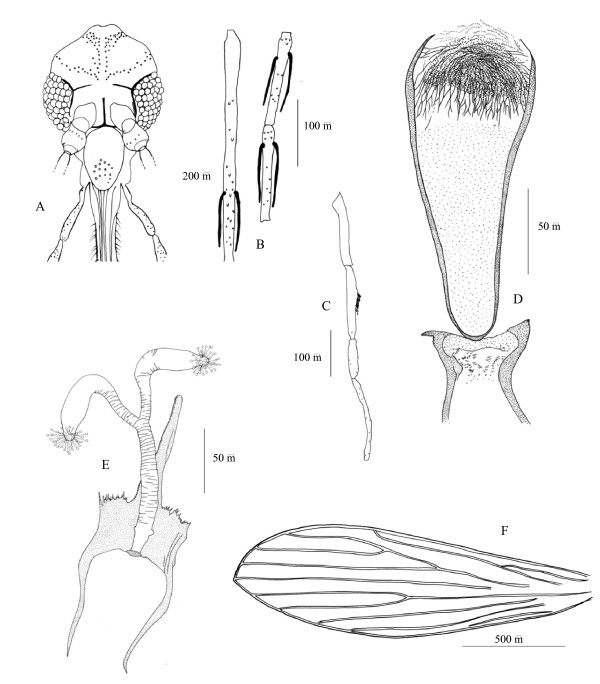
***P. barguesae *n. sp. female**. A: head; B: 3^rd^, 4^th ^and 5^th ^antennal segments; C: palp; D: pharynx and cibarium; E: furca and spermathecae; F: wing.

8 specimens have been investigated and measured.

#### • Head

- Inter-ocular suture: incomplete.

- Cibarium with denticles toward centre and back. No pigmented patch.

- Pharynx: length = 170 to 240 μm. Typical armature of the subgenus *Euphlebotomus *with two kinds of teeth: medio-anterior long, dark, and pointing toward the front; latero-posterior short, in a semi-circular disposition.

- Palpal formula: 1, 4, 2, 3, 5. About 10 club-like Newstead spines implanted in the middle of the third palpal article

- Ascoid formula: 2/III-XIV; 1/XV. AIII = 350–390 μm. AIV = 140–155 μm. AV = 140–155 μm. AIII/AIV+AV = 1.20–1.30. Long ascoids, doesn't reaching the next articulation.

- Labrum = 250–315 μm. AIII/labrum = 1.30–1.40.

- Eyes consisting of about 45–60 ommatidial facets.

#### * Thorax

- 3–6 antero-inferior mesanepistal setae.

- Wings: Length = 1950–2350 μm; width = 570–780 μm. Pi: positive.

- Long anterior, median and posterior legs measuring a similar total lengh (4 mm) without spines.

#### * Genitalia

- Spermatheca:smooth, with the head not individualised.

- Individual sperm ducts: no frontier between spermatheca and duct. Duct + spermatheca = 178 – 240 μm long. Common duct = 100–130 μm with a wide basal diameter.

##### Derivatio nominis

This species is dedicated to our colleague Maria Dolores Bargues Castello. All specimens have been mounted on individual slides. One holotype female and two paratypes have been deposited in the Museum national d'Histoire naturelle de Paris (France). The other paratypes are available on request at the laboratory of Parasitology of the Faculty of Pharmacy of Reims, France. They have been caught by Philippe Barbazan in February, 2005. In accordance with section 8.6 of the ICZN's International Code of Zoological Nomenclature, we have deposited copies of this article at the following five publicly accessible libraries: Natural History Museum, London, UK; American Museum of Natural History, New York, USA; Museum National d'Histoire Naturelle, Paris, France; Russian Academy of Sciences, Moscow, Russia; Academia Sinica, Taipei, Taiwan.

## Discussion

**Figure 3:**
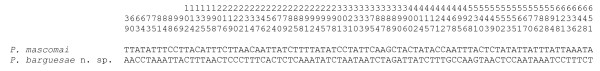
**sequence alignment showing the 86 variable sites between sequences of *P. barguesae *n. sp topotypes and *P. mascomai *topotypes**. Sequence length = 689 bp.

The male-female gathering in the same taxon is based on ecological (cave dwellers caught the same night, in the same place), morphological and molecular criteria. Length of male genital filaments is in agreement with length of females individual sperm ducts, and the width of males aedeagus with width of females common duct. The sequence length is 689 bp for each specimen. Molecular analysis showed 100% homology between cytochrome c oxidase I sequences of males and females [Genbank accession numbers FJ348734–FJ348740 for *P. barguesae *n. sp. and FJ493545 to FJ493548 for *P. mascomai*]. Despite the fact that this marker is commonly sequenced for the "barcode of life" program [13], it has been surprisingly used only once for Phlebotomine sand flies when Arrivillaga *et al*. [14] have characterised several cryptic species within the neotropical complex *Lutzomyia longipalpis*. It has also be used successfully to distinguish two closely related species from North Africa: *P*. (*Paraphlebotomus*) *chabaudi *Croset, Abonnenc & Rioux, 1970 and *P*. (*Pa*.) *riouxi *Depaquit, Killick-Kendrick & Léger, 1997 [15]. This marker strongly supports that the specimens sequenced belong to the same species: *P. barguesae *n. sp. The figure 3 shows the high variability between sympatric *P. barguesae *n. sp. and *P. mascomai *topotypes.

The differential diagnosis with other species of *Euphlebotomus *is easy for both sexes. The females have exclusive smooth spermathecae and the males have a blunt-end aedeagus not previously observed in the subgenus *Euphlebotomus*.

In 1948, Theodor created within the genus *Phlebotomus*, the sub-genus *Euphlebotomus*, with *P. argentipes *as type-species, that he individualized on the following states of characters [16]:

for males: – style with 5 spines, similar to that of *Larroussius*

- paramere trilobed, with or without an accessory spine,

- aedeagus short and conical.

for females: – pharynx with median armature of small teeth and posterior parallel ridges,

- spermathecae segmented or striated with apical segment defined and enlarged.

The male specimens of *P. barguesae *n. sp. can be easily included into the Theodor's definition. However, if the females of *P. barguesae *n. sp. have a pharyngeal armature typical of the subgenus, the smooth spermathecae differ from the Theodor's definition. Despite this disagreement, we include *P. barguesae *n. sp. within the subgenus *Euphlebotomus *containing at the present time 13 taxa including this new species, all Asiatic: *P. argentipes*, vector of *Leishmania donovani *in India, *P. automnalis *Artemiev, *P. caudatus *Artemiev, *P. kiangsuensis *Yao & Wu, *P. lengi *Zhan, He et Ward, *P. mascomai*, *P. mesghalii *Seyedi-Rashti & Nadim, *P. nadimi *Javadian, Jalali-Galousang & Seyedi-Rashti, *P. philippinensis philippinensis *Manalang, *P. philippinensis gouldi *Lewis, *P. tumenensis *Wang & Chang, and *P. yunshengensis *Leng. If we consider i) the inclusion of *P. lengi *within the subgenus *Euphlebotomus *needs further studies, ii) the sharing of the remarkable character "presence of a paramere spine" with some *Anaphlebotomus *species, iii) the definition doesn't covering the morphological patterns of spermathecae, and iv) the variable number of appendices of the paramere, a major revision of the "*Euphlebotomus-Anaphlebotomus" *group has to be done at the light of both molecular and morphological studies. Waiting this study, we consider *P. barguesae *n. sp. as belonging to the subgenus *Euphlebotomus*.

## Conclusion

*P. barguesae *n. sp. is a new species of Phlebotomine sandflies. The male-female gathering in the same species is strongly supported by ecological, morphological and molecular criteria. We consider this new species belongs to the subgenus *Euphlebotomus *(male having 5 spines on the style and a trifurcated paramere, female pharyngeal armature). However, the original smooth spermathecae opens a discussion on the heterogeneity of this subgenus.

## Competing interests

The authors declare that they have no competing interests.

## Authors' contributions

JD, FM and NL participated in morphological analysis of the specimens. JD and FM did drawings, measurements and molecular biology. JD, FM and NL did interpretation of data and drafted the manuscript. All authors read and approved the final copy of this manuscript.
